# When heat casts a spell on the DNA damage checkpoints

**DOI:** 10.1098/rsob.140008

**Published:** 2014-03-12

**Authors:** Thomas Turner, Thomas Caspari

**Affiliations:** Genome Biology Group, College of Natural Sciences, School of Biological Sciences, Bangor University, Brambell Building, Deiniol Road, Bangor, Wales LL57 2UW, UK

**Keywords:** hyperthermia, ATR, ATM, Chk1, Chk2, DNA damage checkpoint

## Abstract

Peregrine Laziosi (1265–1345), an Italian priest, became the patron saint of cancer patients when the tumour in his left leg miraculously disappeared after he developed a fever. Elevated body temperature can cause tumours to regress and sensitizes cancer cells to agents that break DNA. Why hyperthermia blocks the repair of broken chromosomes by changing the way that the DNA damage checkpoint kinases ataxia telangiectasia mutated (ATM) and ataxia telangiectasia and Rad3-related (ATR) are activated is an unanswered question. This review discusses the current knowledge of how heat affects the ATR–Chk1 and ATM–Chk2 kinase networks, and provides a possible explanation of why homeothermal organisms such as humans still possess this ancient heat response.

## 2. Introduction

In the second half of the nineteenth century, the American surgeon Coley [1] noted the curious case of Fred Stein whose tumour disappeared following a high fever from a bacterial infection. Other medical pioneers, including Robert Koch, Louis Pasteur and Emil von Behring, recorded similar cases in which tumour regression coincided with an episode of high body temperature. The use of inactivated bacteria to induce a fever in cancer patients became known as Coley's toxin and proved very successful until it was superseded by the introduction of radiotherapy. Almost a century later, researchers in the 1970s rediscovered that cancer cells arrested cell cycle progression in S and G2, underwent cell death and, most unexpectedly, became unable to repair broken chromosomes after a short exposure (approx. 30 min) to temperatures between 41°C and 45°C [2–4]. Why elevated temperatures block the removal of DNA double-stranded breaks (DSBs) is still not well understood despite the successful use of heat to sensitize cancer cells to radiotherapy [5]. This review summarizes the current knowledge of how hyperthermia reprogrammes the cellular responses to DNA DSBs and offers a possible answer to why human cells still possess this ancient response.

## 3. The DNA damage checkpoint pathways

**Figure 1.**
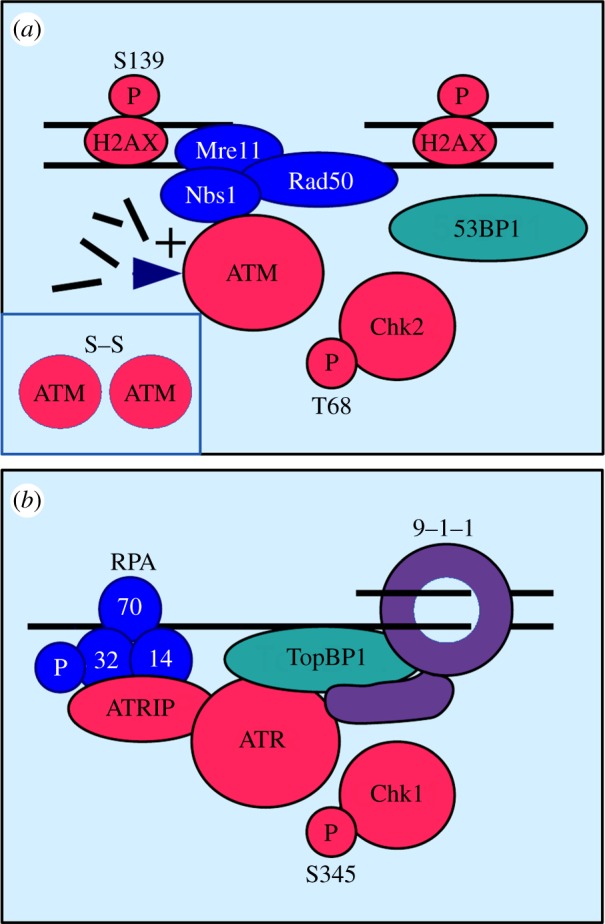
Activation of ATM and ATR at 37°C. (*a*) Activation of ATM. The Mre11–Rad50–Nbs1 (MRN) complex recruits ATM kinase to a broken chromosome. Association of ATM with Nbs1 and the release of short DNA fragments by the Mre11 nuclease within the MRN complex activate ATM. Inactive ATM is a homodimer linked by a disulfide bond (inset). Active, monomeric ATM phosphorylates Chk2 kinase at threonine-68 (T68) and the histone-variant H2AX at serine-139 (S139). Dependent on the cell cycle stage, 53BP1 regulates the conversion of a blunt-ended break into a break with single-stranded DNA tracks. (*b*) Activation of ATR. The ATRIP subunit directs ATR kinase to single-stranded DNA covered by the trimeric replication protein A (RPA). Activation of ATR requires the independent recruitment of the Rad9–Rad1–Hus1 ring (9–1–1) and TopBP1. The tail domain of Rad9 and the ATR-activation domain of TopBP1 both stimulate ATR kinase. Active ATR phosphorylates Chk1 kinase at serine-345 (S345) and the RPA32 subunit or RPA.

A network of kinases, known as the DNA damage checkpoints, respond to broken DNA in all eukaryotic cells. The sensor kinases ataxia telangiectasia mutated (ATM)^Tel1^ and ataxia telangiectasia and Rad3-related (ATR)^Rad3,Mec1^ are directly recruited to different DNA structures at a chromosomal break (reviewed in [6,7]). While ATM binds to the Mre11–Rad50–Nbs1 (MRN) complex at a blunt-ended DSB which lacks long overhangs, the association of ATR with a broken chromosome requires the conversion of the blunt ends into long tracks of single-stranded DNA (ssDNA; figure 1).

The interaction of ATM with Nbs1 and the production of small DNA fragments by the Mre11 nuclease of the MRN complex jointly activate the kinase. Both events trigger the dissociation of the inactive ATM dimer and the phosphorylation of the effector kinase Chk2 at threonine-68 by the active ATM monomers [8]. Another important target of ATM is the histone variant H2AX which becomes phosphorylated at serine = 139 along extensive stretches on either side of the break (figure 1*a*) [9]. The modification of γ-H2AX amplifies the signal by attracting more proteins such as 53BP1^Crb2,Rad9^ to the DNA lesion. 53BP1 plays an important role at the break as it coordinates its conversion into ssDNA depending on the cell cycle stage [10]. This conversion is suppressed in G1 when breaks are rejoined without extensive processing by the non-homologous end-joining pathway. Once cells have copied the chromosomes in S phase, 53BP1 permits end resection to allow for the repair of a subset of DSBs by homologous recombination in G2 (reviewed in [11–13]). The MRN complex contributes to this end resection process by opening up the break site for DNA processing enzymes [14]. Subsequently, ATR kinase binds to the trimeric replication protein A (RPA) on the ssDNA via its subunit ATR interacting protein (ATRIP). After the independent recruitment of the scaffold protein TopBP1^Rad4^ and the Rad9^Ddc1^–Rad1^Rad17^–Hus1^Mec3^ (9–1–1) ring to the damaged site, ATR becomes active and phosphorylates its downstream effector kinase Chk1 at serine-345 (figure 1*b*).

While both kinases provide a robust defence against the malignant potential of DSBs at 37°C, their changed activation under heat stress conditions seems to impair the repair of broken chromosomes. This is especially surprising as heat is a potent activator of ATR and ATM.

## 4. Heat activation of the ATR–Chk1 kinase cascade

**Figure 2.**
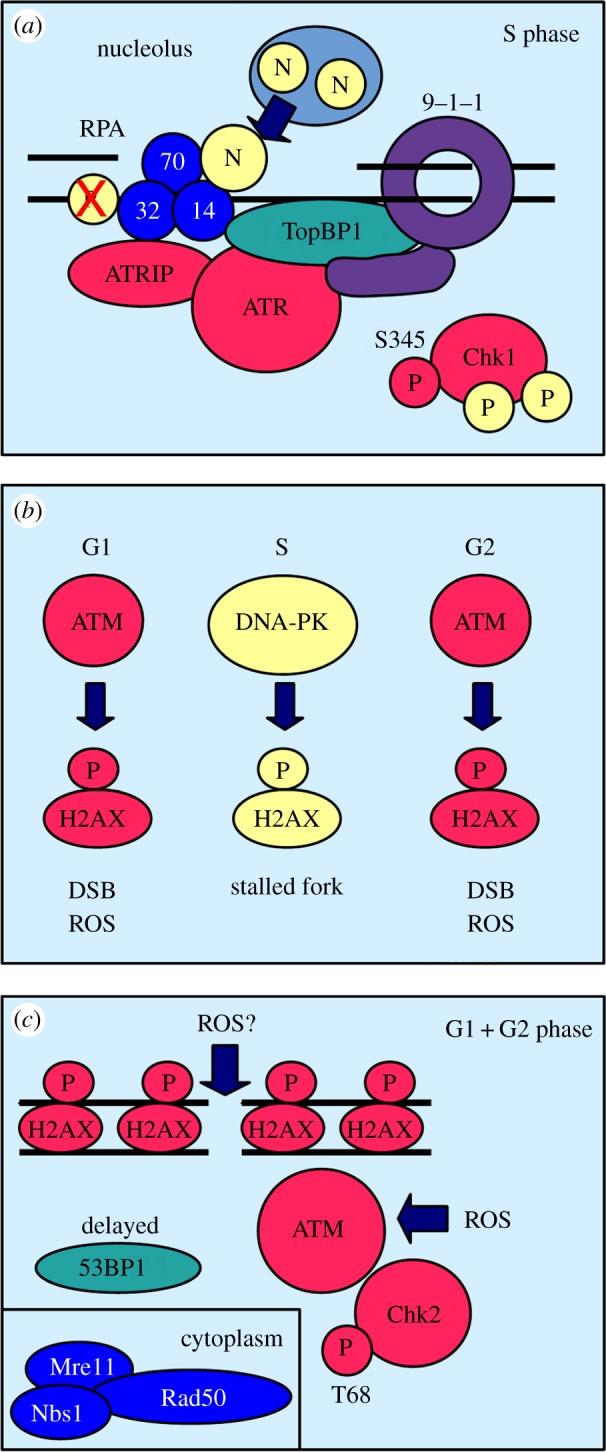
Activation of ATM and ATR at temperatures above 40°C. (*a*) Activation of ATR. Nucleolin (N) binds to replication protein A (RPA) after its heat-induced release from the nucleolus. This reprograms ATR activation. Despite the presence of the Rad9–Rad1–Hus1 ring (9–1–1) and TopBP1, heat-activated ATR does not modify RPA32. Chk1 is phosphorylated at several sites in addition to serine-345 (S345). (*b*) Cell cycle specificity of histone H2AX phosphorylation. In G1 and G2, ATM kinase phosphorylates the histone variant H2AX in response to DSBs or high levels of reactive oxygen species (ROS). During S phase, the related kinase DNA-PK modifies H2AX to stabilize stalled replication forks. (*c*) Activation of ATM. The Mre11–Rad50–Nbs1 complex moves from the nucleus to the cytoplasm (inset) in the response to heat stress. Despite the absence of the MRN complex, ATM kinase is activated in a yet unknown way by heat and phosphorylates Chk2 kinase at threonine-68 (T68). The recruitment of 53BP1 is delayed. Heat, inflammation and increased metabolism increase the levels of ROS which can open the disulfide bond of the inactive ATM dimer thereby activating ATM. DNA double-strand breaks may be a secondary consequence of high ROS levels.

One of the earliest signs of heat stress is the rapid phosphorylation of human ATR at serine-428 and Chk1 at serine-345 (S345) (figure 2*a*) [15,16]. While ATR activation by heat and DNA damage clearly overlaps, some important features are very different. As in the case of DNA damage, Chk1 phosphorylation at S345 by ATR requires the presence of the 9–1–1 ring and the recruitment of the scaffold protein TopBP1 [17]. However, ATR does not modify the subunit RPA32 of RPA as it does in the case of DNA damage at 37°C, and its heat activation is not associated with the mono-ubiquitinylation of Fanconi anaemia group D2 protein (FancD2) [17]. The attachment of one ubiquitin moiety to FancD2 results in its localization to DNA lesions where it contributes to homology-directed DNA repair at 37°C [18]. Moreover, human Chk1 becomes phosphorylated at sites other than S345 at elevated temperatures [16].

Heat activation of ATR–Chk1 signalling arrests cell cycle progression at the G2/M boundary and reduces the rate of cell death [15]. This antagonistic relationship between the cell cycle arrest and apoptosis implies that heat produces chromosomal lesions which trigger cell death if they are not removed before the onset of mitosis. Interestingly, Chk1 is also required for a heat-induced G2/M arrest in the fission yeast *Schizosaccharomyces pombe* [19,20], but unlike the human protein, *S. pombe* Chk1 becomes dephosphorylated at S345 in a process linked with the heat-induced production of an N-terminally truncated variant of the 9–1–1 subunit Rad9 [20]. Why human Chk1 is modified whereas yeast Chk1 becomes dephosphorylated is not yet clear, but this difference may be related to the onset of heat-induced apoptosis in multicellular organisms.

Given the central role of ssDNA in the activation of ATR at 37°C [21], the rapid stimulation of this kinase at temperatures above 40°C could be seen as an indicator of DNA damage. Whether heat produces DSBs is still a matter for discussion. Recent work by Velichko *et al.* [16] showed that heat can indeed cause DSBs, but only in G1 and G2, and not in S phase (table 1). This cell cycle profile of heat-induced DSBs is quite peculiar as human cells are much more heat sensitive in S phase compared with G1 and G2 [3,22]. The absence of DSBs in S phase could be caused by the ability of heat to slow down or stop the extension of replication forks [16,23,24]. This heat-induced replication arrest coincides with the relocalization of important replication proteins such as proliferating cell nuclear antigen (PCNA) to the nuclear matrix [24] and the rapid release of the nucleolin from the nucleolus [25,26] (figure 2*a*). Importantly, nucleolin may act as a signalling protein under these conditions as it returns to the nucleolus within the first 2 h after a heat shock, whereas DNA replication remains arrested for up to 8 h [25]. Because nucleolin and ATRIP both bind to RPA, the release of this protein from the nucleolus may switch ATR activation from its DNA damage to its heat stress mode (figure 2*a*).

**Table 1.** Cell cycle specific heat responses of human cells. γ-H2AX refers to the phosphorylation of the histone variant H2AX at serine-139. DSBs, DNA double-stranded breaks; SSBs, DNA single-stranded breaks; sensitivity refers to the frequency of cell death in the different cell cycle stages.

	G1	S	G2
γ-H2AX	low	high	low
DSBs	yes	no	yes
SSBs	no	yes	no
sensitivity	low	high	low

Although high temperatures do not result in DSBs during S phase, human cells quickly accumulate single-stranded breaks (SSBs; table 1) [16]. This increase in SSBs could be a consequence of the rising number of replication intermediates at stalled forks or it could be an indirect result of the removal of oxidized nucleotides by base excision repair. As discussed later, heat stress increases the levels of reactive oxygen species (ROS) in cells which could lead to a rise in DNA modifications and breaks. The accumulation of SSBs may also be responsible for the strong increase in phosphorylated H2AX upon a heat shock as this modification can occur at ssDNA regions that arise during replication stress or repair, and thus not solely correlates with DSB formation [27]. This may also explain why several groups reported the appearance of phosphorylated H2AX after a heat shock without being able to detect DSBs [28–30].

Taken together, the rapid increase in ATR–Chk1 signalling may protect DNA replication forks that slow down or stop at elevated temperatures. The structure of such forks may however differ from forks that stall at 37°C, explaining the changes to ATR activation.

## 5. Heat activation of the ATM–Chk2 kinase system

The strong increase in γ-H2AX phosphorylation, especially in heat-treated S phase cells (table 1), points towards ATM as the responsible kinase given its importance at 37°C (figure 1*a*) [9]. Consistent with an earlier report [31], ATM modifies H2AX at elevated temperature but only in G1 and G2 cells, whereas the closely related kinase DNA-dependent kinase (DNA-PK) phosphorylates the variant in S phase (figure 2*b*) [16]. This is an interesting observation as DNA-PK normally plays an important role in the repair of DSBs by non-homologous end-joining [32]. The requirement of DNA-PK, but not of ATM, during S phase is very intriguing especially because heat does not cause DSBs during this cell cycle stage (table 1). Taken together, these observations paint a different picture of ATM activation under heat stress conditions. ATM may phosphorylate H2AX at broken chromosomes in G1 and G2, whereas DNA-PK modifies the histone variant at arrested replication forks (figure 2*b*). Consistent with this conclusion, heat-induced phosphorylation of γ-H2AX stabilizes arrested forks [16] and is not linked with an increase in cell death [33].

As in the case of ATR, heat activation of ATM is different from its stimulation by DSBs at 37°C (figure 2*c*). First, the MRN complex, which is crucial for its recruitment to DSBs at 37°C, relocalizes from the nucleus to the cytoplasm at high temperatures [34]. Second, ATM activation is normally accompanied by the association of 53BP1 with the broken chromosome, where the scaffold protein regulates the formation of ssDNA depending on the cell cycle stage [10]. Heat strongly suppresses the recruitment of 53BP1 to DNA [28,35], implying that ATM may not only signal the presence of DSBs. Finally, ATM activation is delayed by 1–2 h at temperatures above 40°C relative to ATR, although ATM acts before ATR when DNA breaks at 37°C [15].

This curious delay could be caused by the slow conversion of SSBs to DSBs when heat-stressed cells progress from S phase into G2, or it could be an indirect effect of a rise in oxidative stress at elevated temperatures. Work in animals revealed an increase in tumour blood flow and oxygen delivery at temperatures of up to 42°C [36], and *in vitro* experiments demonstrated the generation of ROS and 8-oxoguanine formation in DNA under heat shock conditions [37]. An increase in ROS would provide an elegant explanation for the unusual activation of ATM kinase under hyperthermic condition as ROS have been shown to act directly on the inactive ATM dimer by opening a disulfide bond between the monomers without the requirement of the MRN complex [38] (figure 2*c*).

On balance, the slow stimulation of ATM–Chk2 signalling at elevated temperatures may well be triggered by a rise in ROS rather than by the accumulation of DSBs. If the appearance of DSBs in G1 and G2 are a delayed consequence of high ROS levels, then ATM would already be active by the time the DNA breaks thus explaining its activation in the absence of the MRN complex.

## 6. Why do human cells fail to repair double-strand breaks although ATR and ATM are active?

Stimulation of ATR and ATM by heat is clearly distinct from their activation by DNA damage at 37°C (figure 2). Given that heat is a potent replication inhibitor, ATR may help to stabilize arrested forks rather than signalling DSBs. This notion is supported by the finding that an artificial delay of S phase progression using the DNA polymerase-alpha inhibitor aphidicolin suppresses the heat sensitivity of human cells [24]. The release of nucleolin from the nucleolus may allow ATR to distinguish between normal and heat-induced replication problems (figure 2*a*). This distinction may be important, because replication forks which stall at high temperatures may possess distinct DNA structures. The modification of H2AX by DNA-PK may therefore help to stabilize such forks to prevent cell death and the accumulation of DSBs.

Although hyperthermia can cause DSBs in G1 and G2, activation of ATM may be more closely related to the response to ROS as its upregulation is independent of the MRN complex. This shift in activity may simply reflect the higher impact of ROS on cell survival under heat stress conditions. Tissue damage linked with an increase in ROS was, for example, observed in the intestine of mice after a mild heat episode [39].

In summary, the new roles of ATR and ATM at elevated temperatures seem to require changes to their activation mechanisms which are not compatible with their normal functions at broken chromosomes. This would render human cells sensitive to agents that break DNA at temperatures above 40°C.

## 7. Why was the heat response not lost during the evolution of homeotherm organisms?

**Figure 3.**
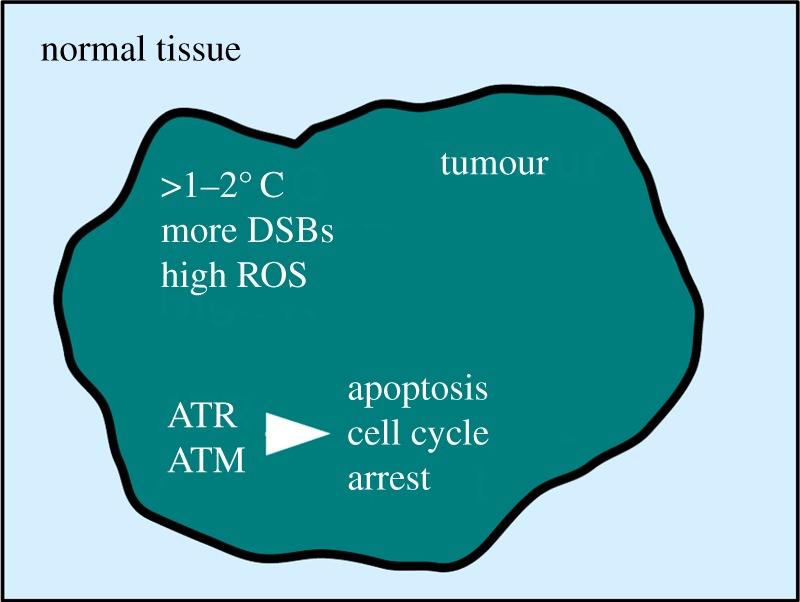
The anti-cancer function of heat activation of ATM and ATR. Elevated temperatures inside solid tumours and increased levels of reactive oxygen species (ROS) reprogramme ATR and ATM signalling. This impairs the repair of DNA double-stranded breaks (DSBs) which accumulate in quickly dividing cancer cells. ATR and ATM cause tumour regression by inducing cell death (apoptosis) and by imposing a cell cycle arrest. At the same time, normal tissue is protected.

A possible answer to this riddle may lie in the case of Fred Stein whose sarcoma regressed in the response to a fever [1]. Up to 4% of all cancer patients develop metastasis (secondary tumours) without having a primary tumour, a phenomenon known as cancer of unknown primary origin [40]. Of course, the primary malignancy may have been too small to be detected, but, alternatively, the primary tumour may have regressed prematurely. Driven by high metabolism, inflammation and blood perfusion, the temperature of bladder tumours is up to 2°C higher when compared with the temperature of the surrounding tissue [41]. Similar observations were made with breast cancer patients where the skin temperature above the lesion can be between 1°C and 2°C higher [42]. Because malignant cells do possess a high level of DSBs [43], the inability of ATR and ATM to respond to DNA breaks at elevated temperatures may be advantageous as it would cause their death by apoptosis (figure 3). Moreover, the ability of the ATR–Chk1 pathway to arrest cells in G2 at high temperatures would also act against quickly dividing cancer cells. Unfortunately, most tumours breach this natural defence by inactivating the cell death pathways [44], rendering the spontaneous regression of tumours a rare event.

## 8. Conclusion

By shifting the activity of the ATR–Chk1 and ATM–Chk2 pathways from DNA damage to heat sensing, evolution may have ‘hit two birds with one stone’. While normal cells are protected from the consequences of heat-induced replication arrests and ROS, malignant cells may fall victim to the high number of broken chromosomes which cannot be repaired at elevated temperatures (figure 3). A deeper understanding of ATM and ATR activation at high body temperatures will pave the way for novel therapies to tackle solid tumours. Peregrine Laziosi clearly benefited from this natural response in the fourteenth century, because the spontaneous regression of his tibia tumour not only saved his leg but also made him a saint.
